# New Tools for Conservation Biological Control: Testing Ant-Attracting Artificial Nectaries to Employ Ants as Plant Defenders

**DOI:** 10.3390/insects11020129

**Published:** 2020-02-17

**Authors:** Enrico Schifani, Cristina Castracani, Daniele Giannetti, Fiorenza Augusta Spotti, Roberto Reggiani, Stefano Leonardi, Alessandra Mori, Donato Antonio Grasso

**Affiliations:** 1 Department of Chemistry, Life Sciences & Environmental Sustainability, University of Parma, Parco Area delle Scienze, 11/a, 43124 Parma, Italy; daniele.giannetti@unipr.it (D.G.); fiorenzaaugusta.spotti@unipr.it (F.A.S.); stefano.leonardi@unipr.it (S.L.); alessandra.mori@unipr.it (A.M.); donato.grasso@unipr.it (D.A.G.); 2 Azienda Agraria Sperimentale Stuard, Strada Madonna dell’Aiuto, 7/a, 43126 San Pancrazio, Parma, Italy; r.reggiani@stuard.it

**Keywords:** Integrated Pest Management (IPM), Conservation Biological Control (CBC), ant-plant relationships, multitrophic interactions, applied myrmecology, agroecology, mutualism, indirect defense, *Pyrus* orchard, plant health, pear trees

## Abstract

Knowledge of the role of ants in many agroecosystems is relatively scarce, and in temperate regions the possibility to exploit ants as biocontrol agents for crop protection is still largely unexplored. Drawing inspiration from mutualistic ant–plant relationships mediated by extrafloral nectaries (EFNs), we tested the use of artificial nectaries (ANs) in order to increase ant activity on pear trees and to evaluate the effects on the arthropods, plant health and fruit production. While EFNs secrete a complex solution mainly composed of sugars and amino acids, ANs were filled with water and sucrose only. The results suggest that ANs can be used as manipulative instruments to increase ant activity over long periods of time. High ant activity was significantly linked to lower incidence of the pathogen fungus *Venturia pyrina* (pear scab) on pear leaves, and of the presence of *Cydia pomonella* (codling moth) caterpillars on pear fruit production. These results further encourage exploring underrated possibilities in the development of new tools for conservation biological control (CBC).

## 1. Introduction

Plants have evolved very complex relationships with ants, and have been fundamental to the rise of many modern lineages of these insects [1,2,3,4]. Some of these relationships are strictly antagonistic, revolving around herbivory in a few New World species and more commonly around seed predation [5]. Many of the other relationships are beneficial to plants, and encompass several different aspects from seed dispersal and soil processing to rare cases of pollination [6,7], while the vast majority are based on an ant’s appetite for sugary liquids [1], which is produced in the form of honeydew by sap-feeding insects (mainly heteropterans) or directly by plants in the form of nectars. Ants may protect sap-feeding insects in exchange for honeydew [8,9,10,11]. Although ants may indirectly damage plants through this relationship, its cost–benefit ratio may still be beneficial overall for the plant if ant presence displaces more damaging herbivores [12]. Some plants produce nectars, other food rewards or offer shelter to ants in exchange for protection against herbivory, competing plants or even pathogens [13,14,15,16,17]. These rewards may also be aimed at distracting ants from tending sap feeders [18,19,20,21]. Several cases of these ant-plant mutualisms are mediated by extrafloral nectaries (EFNs), which have been described for 4017 plant species of over 450 evolutionary lineages (21% of vascular plant families), and are expected to be found in about 8000 species [22,23]. EFNs are both a food reward for ants and manipulative tools to maximize plant advantages in the ant–plant relationship [24,25,26]. They produce complex solutions in which sugars and amino acids are the main components, while less abundant constituents such as secondary metabolites may also play an important role [25,26]. Moreover, in some plants EFNs can be an induced defense in response to herbivory [27]. However, plant defense by ants against herbivores can also be mediated by other adaptations, such as certain volatiles [28,29,30,31,32]. Although the latter mechanism has only been documented in a few cases, it is easy to speculate that ants may act as plant defenders by attacking herbivores much more often than currently reported.

An urgent need to develop more-sustainable agriculture is widely recognized, and Integrated Pest Management (IPM) should reduce the use of pesticides by favoring biological control techniques [33,34,35]. The use of ants as biocontrol agents is overlooked in comparison to other insect groups, and their use has been limited mainly to equatorial and tropical regions. Nonetheless, the oldest case of biological control mediated by insects (304 A.D.) attests to the use of the ant genus *Oecophylla* Smith, F., 1860 as a biocontrol agent [36]. Today, *Oecophylla* spp. are still used in more than 20 countries of Africa, Asia and Oceania to control about 50 different pest species [37,38]. Although they are considered bioindicators of soil function and habitat quality in rural environments [39,40], both their distribution and their role in most agroecosystems are still insufficiently documented. However, ants have several characteristics that make them good candidates for biocontrol agents [5,38,41,42,43,44,45,46]. First, they may be generalist predators, whose polymorphism often enhances their polyphagy. Second, many ants are often territorial and aggressive, thus chasing away intruders unsuitable as prey. In addition, recruitment allows a quick reaction to increases in prey. Moreover, colonies can withstand periods of food shortage and without becoming particularly susceptible to satiety because they build food stocks and rear immature stages. Finally, ant activity in agroecosystems can be easily manipulated, for example, by transferring colonies or offering additional food sources and nest sites (e.g., [47,48]). In addition, the manipulation of ant foraging pathways may redirect foragers towards target pests, and can be conducted using plants with EFNs [49].

Ant–sap feeder interactions are usually the main cause of concern about the role of ants in some agroecosystems. For example, in vineyards and citrus orchards, a number of studies have shown that ant presence increases populations of mutualistic aphids and coccids and decreases those of some of their natural enemies (e.g., [50,51,52,53,54,55,56]). As a result, additional research has focused on the control of ant populations in these agroecosystems, either by employing chemical substances (e.g., [57,58,59,60,61,62,63,64]) or distracting tending ants by providing sugary substances (e.g., [21]). The latter method may allow the positive effects of ant presence (see [12]). Nevertheless, other studies have found that the presence of ants in citrus and vine groves was weakly related or not related at all to the presence of sap feeders [21,65]. In addition, some natural predators of aphids and coccids may even increase under ant presence in vineyards [51]. Moreover, not all economically important sap-feeding pests are mutualists, and many ant species are not mutualists either. Even in citrus and vine groves where the phenomenon has attained particular attention, only a small proportion of ant species has been linked to pest outbreaks. In fact, of the 123 ant species inhabiting South Africa citrus orchards, only about 25 tend aphids and only three or four are considered responsible for pest outbreaks [66,67].

In Europe, the use of ants in biological control has mostly developed around wood ants (*Formica rufa* group). These have usually been employed in forest ecosystems [68,69], and much more rarely in agroecosystems, to which they had to be transported [48,70]. Only a few studies have dealt with ants in Italian agroecosystems (e.g., [71,72,73,74,75,76,77]). However, native ant species common in Italy and in the Mediterranean basin could be effective control agents against highly problematic pests such as the exotic brown marmorated stink bug *Halyomorpha halys* (Stål, 1855) [78], fungi and other herbivores [79]. In some cases, common harvester ants may have a positive role in the control of weeds [80]. Many of the above-mentioned ant characteristics considered promising for biological control were also documented for Italian ants (e.g., [7,68,74,75,81,82,83,84]).

Our paper reports on the role of ants and their use in conservation biological control (CBC) [85,86], and our experiment was carried out in a pear agroecosystem. Pear trees have been cultivated for at least 3000 years, and currently continue to be important for fruit production in wide regions of the planet, from Eurasia to North America [87]. Moreover, in Italy, a remarkable diversity of wild and cultivated forms has been documented [88], none of which possess EFNs or other structures aimed at attracting ants. Finally, pear orchards host a rich community of pest insects, whose control costs about USD 14 million each year worldwide, and does not usually employ ants [89]. However, ant presence in pear agroecosystems is not commonly associated with pest outbreaks. Conversely, *Crematogaster subdentata* Mayr, 1877 was reported to suppress a population of the San Jose scale *Quadraspidiotus perniciosus* Comstock, 1881 [90]. Moreover, *Formica neoclara* Emery, 1893 was considered to be a promising biocontrol agent of the pear psylla, *Cacopsylla pyricola* (Foerster, 1848) [91,92,93]. Although other authors suggest some ants may instead favor the latter [94,95], no clear evidence was provided. Finally, there is other evidence suggesting that *Cacopsylla* spp. primary parasitoids are even favored when ants are present, because they are more effective at keeping away the hyperparasitoids [96].

We introduced artificial nectaries (ANs) to study the impact of ants in a pear agroecosystem. Inspired by EFNs, ANs are manipulative tools designed to increase ant activity on trees by dispensing a liquid made of water and sugar to attract ants. Although a few studies have already tested the use of ANs or other food sources to attain agroecological benefits (e.g., [20,21,97,98,99,100]), none of them have studied ant effectiveness in defending plants, focusing instead on distracting ants from mutualistic sap feeders. Thus, the aim of our study was to evaluate both the functionality of ANs and the impact of different levels of ant activity on arboreal arthropodofauna, plant health and fruit production.

## 2. Materials and Methods 

### 2.1. Study Area

Sampling was conducted in a 1-hectare organic orchard located near Pontescodogna, Parma, Italy (44.7378, 10.1954) and part of the Regional Natural Park “Boschi di Carrega”. The orchard consisted of 430 fruit trees (mainly pear, but also apple, apricot, cherry, fig, peach and plum trees), arranged in 15 rows and partially surrounded by a deciduous broadleaved forest. The orchard management was limited to periodical lawn mowing.

### 2.2. Artificial Nectaries (ANs)

**Figure 1:**
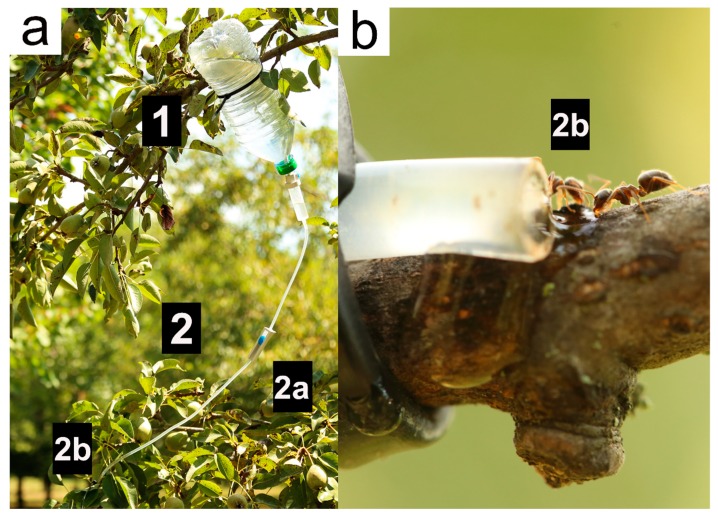
Artificial nectary (AN) installed on a pear tree. In pictures (**a**) and (**b**), the main components of the artificial nectary are shown: the tank (1) and the infusion set (2). The infusion set is made of the flow controller (2a) and the dispenser (2b). In (**b**), two *Lasius niger* workers are seen drinking at the nectar released by the dispenser.

The artificial nectary used in the experiment was made of a 1l plastic bottles (nectary tank) connected to an infusion set (Figure 1). Two holes were drilled in the bottle: one on its basal surface, later used to add or refill the liquid, and one on its cap, to connect the infusion set consisting of a flow controller and a dispenser releasing the liquid at the AN distal end. The AN created a direct slow, steady and adjustable flux of artificial nectar (set at 15–20 drops per minute) to a focus area on the plant (Nectar-releasing points). The content of the ANs consisted of a liquid solution made of 10g of sucrose in 1l of water and was refilled whenever needed (usually once a week) in order to provide an uninterrupted operation.

### 2.3. Treatments

**Figure 2:**
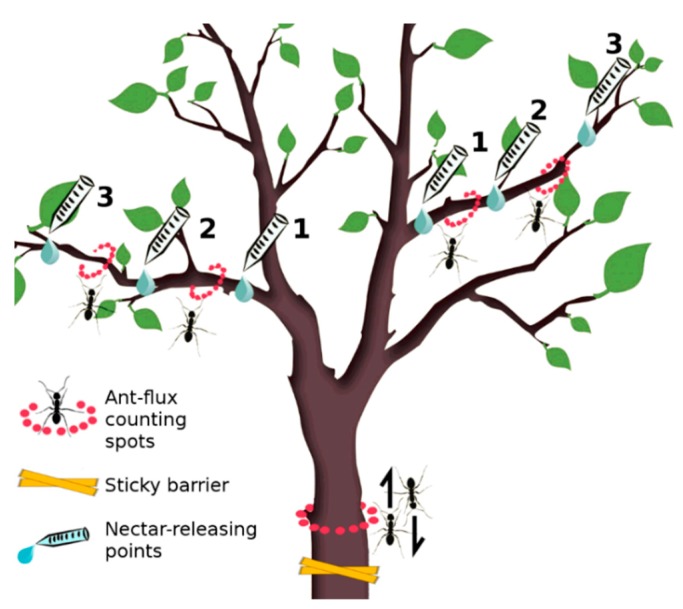
Positioning of the experimental apparatus on the trunk and the two focal branches of the tree. Ant-flux counting spots were present in all the trees (*n =* 20), whereas nectar-releasing points and sticky barriers were present only according to treatments: ANs+ for nectar-releasing points and Ants− for barriers.

A total of 20 adult pear trees, homogenous in size (tree height: 2.5–3 m; trunk Ø: 30–40 cm), were selected for the experiment from two pear rows. They were chosen from among those plants standing far enough away from neighboring trees in order to avoid any contact and eventual passage of ants between them. Four treatment groups, each made up of five randomly selected trees, were then created. On each tree, two of the main branches departing from the trunk were selected as focal branches and used for data collection. Treatment groups differed according to the presence/absence of two different manipulations: ANs and ant-exclusion. Six ANs were placed in each tree in order to create six different release points: three points each per focal branch, one proximally to the trunk, one in the middle of the branch and the last distally to the trunk (Figure 2). Ant-exclusion consisted of the placement of sticky barriers at the base of the trunk in order to prevent soil-nesting ant access to the tree. Sticky barriers are a common system used for ant exclusion in field experiments [20,101,102]. Given the almost complete absence of arboreal-nesting species in the orchard, sticky barriers were expected to be effective in eliminating ants from targeted trees.

As a result, the following treatments were created:

- Treatment 1 (ANs+/Ants+): ANs were installed and ants were free to climb the tree
- Treatment 2 (ANs−/Ants+): no ANs were installed and ants were free to climb the tree (trees with no manipulations)
- Treatment 3 (ANs+/Ants−): ANs were installed and ants were not free to climb the tree since sticky barriers were installed
- Treatment 4: (ANs−/Ants−): no ANs were installed and ants were not free to climb the tree since sticky barriers were installed

### 2.4. Data Collection 

Data were typically collected from 09:00 to 12:00 once a week during a two-month period from the beginning of July to the end of August 2018 (nine weeks). Data recording started one week after the beginning of manipulations.

#### 2.4.1. Ant Activity

Data were collected with the aim of monitoring both abundance and diversity of ants active on the trees according to the four treatments. In order to avoid specimen collection during data sampling, a checklist of ant species in the orchard was compiled before the beginning of the experiment (see Table S1). In June 2018, ants were collected by direct sampling and identified under a ZEISS Stemi 508 stereoscopic microscope. Measurements were taken with the aid of an Axiocam Erc 5 s mounted on the microscope and ZEISS Zen core Software. Taxonomic identifications were mainly made following Radchenko and Elmes [103] and Seifert [104].

For each tree, five ant-flux counting spots were selected to record ant activity: on the trunk (50 cm above the ground) (1), on each focal branch between the first and second (2, 3) and between the second and the third nectar-releasing points (4, 5). At each spot, the species and number of ants were recorded during one-minute samplings (ant flux: N ants/min). Ants were counted if crossing (both directions) an imaginary circumference of the trunk/branch at each spot (Figure 2).

#### 2.4.2. Arthropod Abundance

In order to evaluate the effect of the presence of ants and ANs on the arthropod fauna, the number of all non-ant arthropods (herein simply “arthropods”) was recorded. When possible, individuals were identified at species rank, and, if necessary, a few individuals were collected from adjacent trees (not involved in the samplings) in order to minimize any possible influence on the experiment. Taxonomic aids to identification were Chinery, Leraut and the website Araneae—Spiders of Europe [105,106,107]. Arthropods were counted and identified on both focal branches of each tree through direct observation, by inspecting each branch from side to side. We decided to concentrate on focal branches since the maximum effect of ANs was expected at this location.

In addition, in order to produce a checklist of the arthropods of the area, data from focal branches were integrated with other direct observations on arthropod presence recorded by chance while working on the experiment.

#### 2.4.3. Leaf Damages

**Figure 3:**
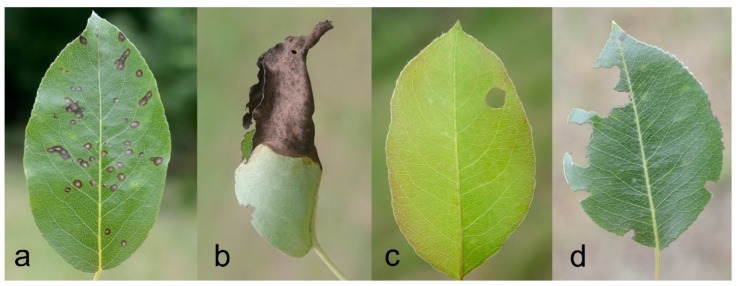
Pear leaves showing the main characteristics of the four damage categories used to evaluate their condition: Scab—S (**a**), Necrosis—N (**b**), Holes—H (**c**), Damaged Edge—DE (**d**). Leaf in picture (**b**) also shows signs of Holes and Damaged Edge.

A preliminary assessment of the most common types of damage affecting the leaves in the study area was conducted during June. These types of damage may be linked to herbivory or pathogens, which are damage sources potentially affected by ant activity (e.g., [48]). As a result, four damage categories were established and a damage scale was set for each category (also see Figure 3):

- Scab (S): presence of distinct black spots on the leaf surface, attributed to the fungus *Venturia pyrina* Aderh. (1896). Scores: 1 = absent; 2 = low (less than half leaf surface interested); 3 = medium (half of the leaf surface interested); 4 = high (more than half leaf surface interested)
- Necrosis (N): presence of extended necrotic areas on the leaf (surfaces larger than spots and with different shapes). Scores: 0 = absent; 1 = present
- Holes (H): presence of holes due to missing parts of tissue far from the edges. Scores: 0 = absent; 1 = present
- Damaged Edge (DE): presence of altered leaf profile due to missing parts of tissue at the edges; Scores: 1 = absent; 2 = low (less than half leaf edge interested); 3 = medium (half of the leaf edge interested); 4 = high (more than half leaf edge interested)

The damage level of the leaf was always inferred by visual inspection. Prior to the beginning of the sampling, 15–25 leaves per focal branch were selected and monitored for the duration of the experiment recording for each sampling, damage category and score.

#### 2.4.4. Fruit Damages

In order to describe fruit damage, four hypothetical damage categories were established:

1. Damage caused by sucking or chewing phytophagous insects (e.g., see [108] for *H. halys*)
2. Holes produced by codling moth *Cydia pomonella* (Linnaeus, 1758) caterpillars (Lepidoptera, Tortricidae)
3. Damage caused by fungi (e.g., *V. pyrina*)
4. Other damage

Immediately after being harvested, all fruits of each tree were counted and their statuses labeled as damaged or not damaged for all categories.

### 2.5. Statistical Analyses

If not specified, data were analyzed using IBM SPSS Statistics software (Italian version 25). Analyses and results are presented according to the nomenclature reported in [109].

In order to test the effect of treatments and time on ant activity, a mean ant flux (*n =* 5) was calculated for each tree and week and was used as the dependent variable in a repeated measures ANOVA (*n =* 180: 5 trees × 4 treatments × 9 weeks). Weeks 1–9 were considered a within-subjects factor (repeated measures design) and treatments (ANs+/Ants+; ANs−/Ants+; ANs−/Ants−; ANs+/Ants−) were used as a between-subjects factor (independent design). For the repeated measures, if assumption for sphericity was violated (Mauchly’s test), Greenhouse-Geisser correction was used. A repeated contrasts procedure was used to compare weeks and Tukey post hoc tests were used for treatments.

To test the effect of treatments on arthropods, and because of a data distribution clearly deviating from normality, a non-parametric approach was chosen. For each recorded taxon, a Kruskal-Wallis test on abundances was run (*n =* 360: 2 branches × 5 trees × 4 treatments × 9 weeks). All possible pairwise comparisons (*n* = 6) with adjusted *p*-values (Bonferroni) were run only in cases of statistically significant results. Taxa with a total number of less than 10 specimens were excluded from the analysis.

Data on leaf damage were treated as follows: for each leaf damage category (S, N, DE and H) a mean score (*n =* 15–25) was calculated for each branch and week and used as a dependent variable in a repeated measures ANOVA (*n =* 360: 2 branches × 5 trees × 4 treatments × 9 weeks). Weeks 1–9 were considered as a within-subjects factor (repeated measures) and treatments (ANs+/Ants+; ANs−/Ants+; ANs−/Ants−; ANs+/Ants−) were used as a between-subjects factor (independent design). For the repeated measures, if the assumption for sphericity was violated (Mauchly’s test), Greenhouse-Geisser correction was used. A repeated contrasts procedure was used to compare weeks and Tukey post hoc tests were used for treatments. 

To test the effect of the treatments on fruit damage, two separate analyses were run: one on data from focal branches, and the other on data from the rest of the whole tree. In the case of focal branches, a generalized linear model (GLM) with binomial error structure (logistic regression) was run on the status of the fruit (damaged vs not damaged) (*n =* 271 fruits with an average of 13.55 fruits per tree ± 1.92 SE). Treatments (ANs+/Ants+; ANs−/Ants+; ANs−/Ants−; ANs+/Ants−) were considered as a fixed factor. For these analyses, the glm() function of the R statistical package was used [110]. The analysis was repeated to include a random factor for Tree-id using the glmer() function of the lme4 R package. 

The same logistic regression model was used for the analysis of fruit damage on the rest of the whole tree (*n =* 7699 fruits with an average of 384.95 fruits per tree ± 81.97 SE). We found no evidence of over-dispersion in either analysis.

## 3. Results

### 3.1. Ant Activity

**Figure 4:**
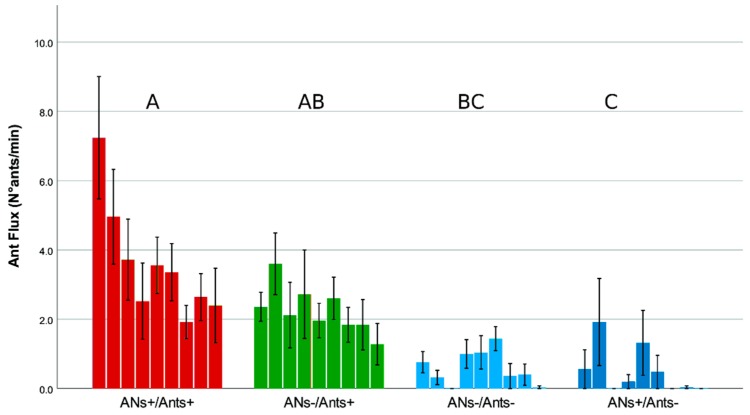
Effects of treatments and weeks on ant flux. Bars represent the mean number of ants crossing (both directions) an imaginary circumference of the trunk/branch at each spot during a single minute. For each treatment, bars correspond to the nine weeks of the experiment (left to right). For each bar, the SE interval is provided. Bars are lumped according to treatment and treatments with the same letter are not statistically different (mixed-design ANOVA and Tukey post hoc tests were used on treatments, see text for further details).

A total of 19 ant species were recorded during the preliminary assessment (Table S1). Among these species, 10 were observed on the experimental trees (Table 1). As concerns the total number of trees where ants were allowed to climb (*n =* 10), *Formica cunicularia, Camponotus piceus* and *Plagiolepis pygmaea* were respectively recorded in 100%, 90% and 70% of the trees. As concerns the ant flux observations (*n =* 450, trunk + branches), the most frequently observed species were *Lasius paralienus*, *Lasius niger* and *F. cunicularia*, which were observed in 22.44%, 16.44% and 12.89% of records, respectively. The most widespread species were *F. cunicularia, C. piceus* and *P. pygmaea*, which were recorded in 100%, 90% and 70% of the trees where ants were allowed to climb (*n =* 10), respectively. The most abundant species were *L. paralienus*, *P. pygmaea* and *L. niger*, with 553, 368 and 335 individuals were counted (*n =* 450), respectively. Only *L. paralienus* and *P. pygmaea* were sporadically able to climb trees despite the sticky barriers (Figure 4).

Data on the “ant flux” revealed a significant main effect of weeks (F_(3.77–60.28)_ = 4.24, *p* = 0.005). Repeated contrasts revealed a general decrease from week 1 to week 9. There was also a significant main effect of treatments (F_(3–16)_ = 11.83, *p* < 0.001). Tukey post hoc tests revealed a decreasing gradient of ant flux from ANs+/Ants+ to ANs+/Ants− treatments.

Results according to treatments and weeks are presented in Figure 4, where differences among treatments are shown, including a decreasing trend from week 1 to week 9 that was mainly evident for the treatments with no ant exclusion (ANs+/Ants+; ANs−/Ants+).

### 3.2. Arthropods Abundance

Arthropods found on the trees during the whole experiment were classified as belonging to 70 taxa: 23 species, 16 genera, 21 subfamilies/families and 10 orders (Table S2). The analysis of arthropod abundance on focal branches revealed the presence of spiders (Arachnida, Aranea) and seven orders of insects: Coleoptera, Dermaptera, Hemiptera, Hymenoptera, Lepidoptera and Neuroptera (Table 2). The most abundant species were *Hyphantria cunea* (Drury, 1773) (Lepidoptera, Erebidae) and *Stephanitis pyri* (Fabricius, 1775) (Hemiptera, Tingidae), but they were only recorded in high numbers during two single events and on trees where ants were not allowed to climb. Over 330 caterpillars of the fall webworm *H. cunea* eliminated almost all the leaves of one focal branch, while about 100 individuals of the pear lace bug *S. pyri* were counted on another. 

As concerns the effect of treatments on arthropod abundances, a Kruskal-Wallis test was performed on the following taxa: Araneae, Chrysopidae eggs, Coleoptera, Diptera, *Vespula* spp. (Hymenoptera, Vespidae), *Metcalfa pruinosa* (Say, 1830) (Hemiptera, Flatidae), *Stephanitis pyri* (Fabricius, 1775) (Hemiptera, Tingidae), other Hemiptera and *Hyphanthria cunea* (Drury, 1773) (Lepidoptera, Erebidae). The remaining taxa were excluded from the analysis due to their low abundances (Table 2). The Kruskal-Wallis test found statistically significant differences in *Vespula* spp. (H_(3)_ = 37.749, *p* < 0.001), *S. piryi* (H_(3)_ = 9.094, *p* = 0.028) and *H. cunaea* (H_(3)_ = 9.050, *p* = 0.029). For *Vespula* spp., pairwise comparisons showed the presence of two groups that differed according to the presence of ANs, with a higher abundance in treatments where ANs were present (Table 2). For *S. piryi* and *H. cunaea*, pairwise comparisons showed no significant differences between groups in all the possible comparisons. Finally, the Kruskal-Wallis tests found no statistically significant differences in Coleoptera (H_(3)_ = 1.759, *p* = 0.624), Araneae (H_(3)_ = 6.268, *p* = 0.099), Diptera (H_(3)_ = 0.727, *p* = 0.867), *M. pruinosa* (H_(3)_ = 3.402; *p* = 0.334), other Hemiptera (H_(3)_ = 2.348, *p* = 0.503) and Chrysopidae eggs (H_(3)_ = 6.642, *p* = 0.084) (Table 2).

### 3.3. Leaf Damage

**Figure 5:**
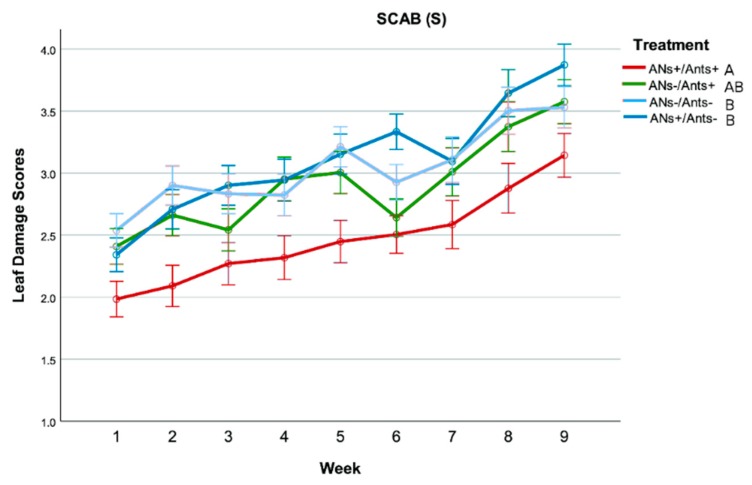
Effects of treatments and weeks on leaf damage for the Scab category. Points represent the mean score per week and treatment. For each point, whiskers show the SE interval. Lines with the same letter are not statistically different (mixed ANOVA and Tukey post hoc tests, see text for further details).

As concerns Scab, there was a significant main effect of weeks on damage scores (F_(3.58–121.82)_ = 51.61, *p* < 0.001). Repeated contrasts revealed a general increase of scores from week 1 to week 9. There was also a significant main effect of treatments (F_(3–34)_ = 4.36, *p* = 0.011). Tukey post hoc tests revealed that ANs+/Ants+ treatment had lower scores than both treatments with ant exclusion (ANs+/Ants− and ANs−/Ants−), which did not differ from one another. ANs−/Ants+ treatment was associated with scores in-between the two previous groups (Figure 5).

**Figure 6:**
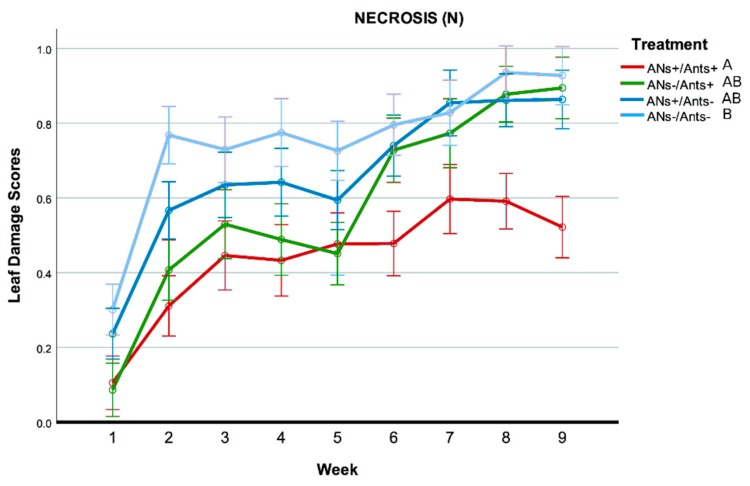
Effects of treatments and weeks on leaf damage for the Necrosis category. Points represent the mean score per week and treatment. For each point, whiskers show the SE interval. Lines with the same letter are not statistically different (mixed ANOVA and Tukey post hoc tests, see text for further details).

The analysis on the Necrosis category showed that there was a significant main effect of weeks on damage scores (F_(5.04–272)_ = 49.00, *p* < 0.001). Repeated contrasts revealed a general increase of scores from week 1 to week 9. There was also a significant main effect of treatments (F_(3–34)_ = 4.39, *p* = 0.010). Tukey post hoc tests revealed that ANs+/Ants+ treatment had lower scores than ANs−/Ants− treatment. ANs−/Ants+ and ANs+/Ants− treatments did not differ from one another and had scores in-between the two previous groups (Figure 6).

As concerns the Damaged Edge category, scores were generally very low with very few records encompassing a score of 2, meaning that damage was absent for less than half the leaf margin. There was a significant main effect of weeks on damage scores (F_(5.89–200.20)_ = 4.48, *p* < 0.001). Repeated contrasts revealed no differences between week 2 and week 8, lower scores for week 1 compared to week 2 and higher scores in week 9 as compared to week 8. No significant main effect of treatments was found (F_(3–34)_ = 0.23, *p* = 0.87). 

The analysis on the Holes category showed generally very low scores with very few records encompassing a score of 0.4, meaning that holes were usually absent. No significant main effects were found on the damage scores of weeks (F_(4.18–142.19)_ = 1.66, *p* = 0.16) and treatments (F_(3–34)_ = 1.12, *p* = 0.36).

### 3.4. Fruit Damage

**Figure 7:**
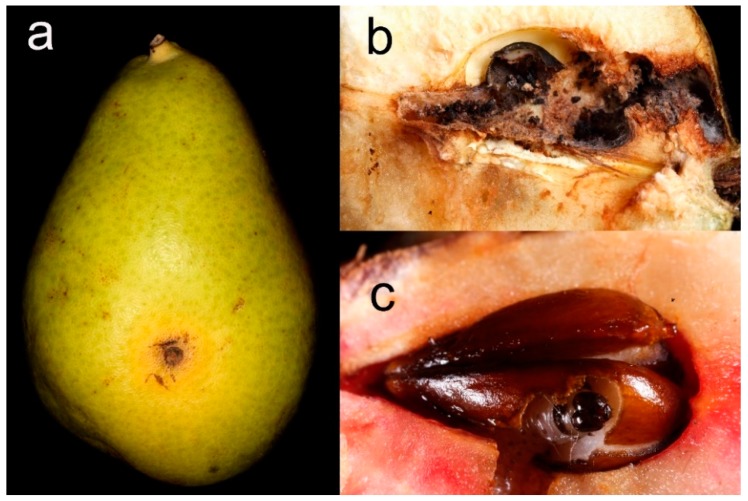
Damage produced by caterpillars of the codling moth *Cydia pomonella* on pear fruits. A small entrance hole (**a**) is usually visible on its side or near the stamen if the fruit is attacked. The larvae penetrate inside, often eating both the pulp and seeds (**b**,**c**), and may create favorable conditions for other organisms such as fungi to grow within the fruit (**b**).

As concerns fruit damage, only holes produced by caterpillars of the codling moth *C. pomonella* were recorded and used for a statistical analysis (Figure 7). Only 3% of the fruits of the whole fruit production from ANs+/Ants+ trees were damaged by *C. pomonella*, whereas this value increased for the other treatments (ANs−/Ants+: 23%; ANs−/Ants−: 28%; ANs+/Ants−: 11%) (Table S3).

**Figure 8:**
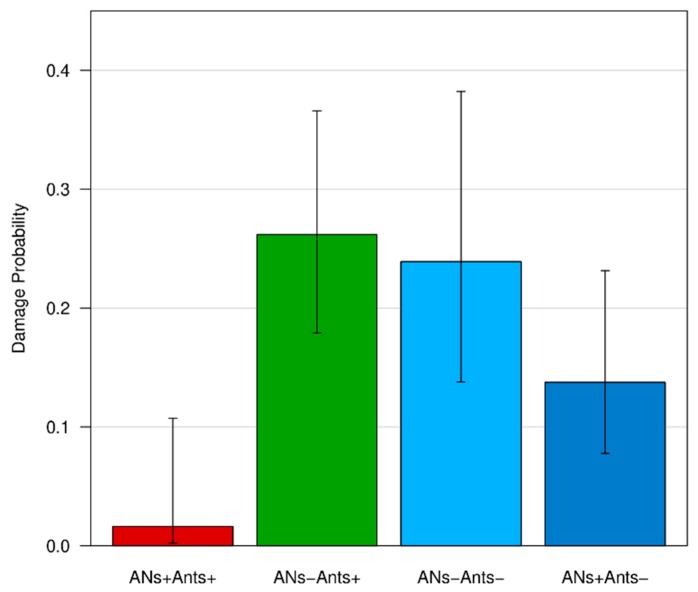
Effects of treatments on fruit damage by *C. pomonella* for focal branches. Bars represent the probability of a fruit to be damaged according to the treatment (logistic regression model). For each bar, a confidence interval is provided.

The logistic regression analysis showed a significant association between treatments and fruit status (damaged vs not damaged) on the focal branches. Fruits from ANs+/Ants+ trees were 10 times less likely to be damaged than the control group (ANs−/Ants+), whereas no differences were detected between the control group and the other treatments (ANs+/Ants−; ANs−/Ants−) (see Table 3 and Figure 8). The addition of the random factor Tree-id to the model did not change previous results.

**Figure 9:**
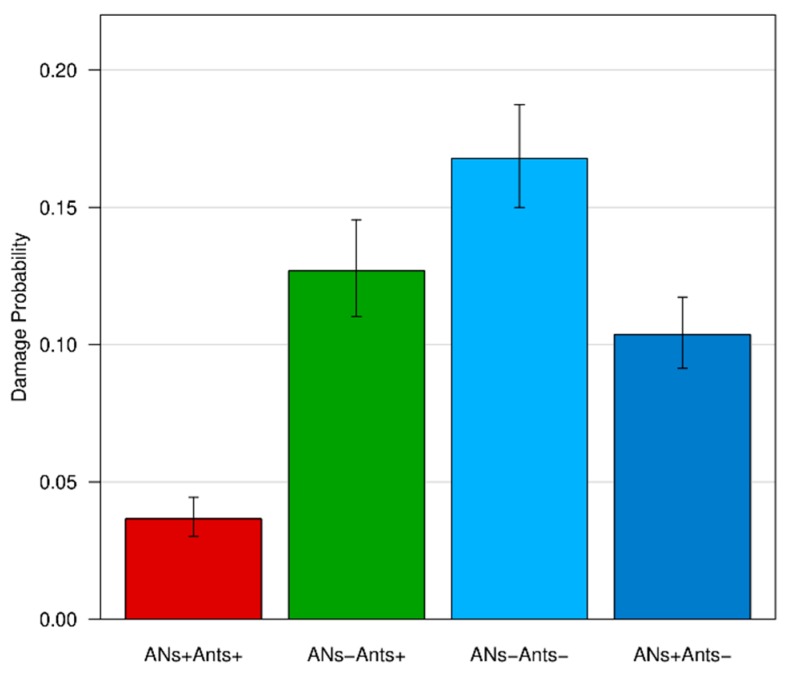
Effects of treatments on fruit damage by *C. pomonella* for the whole tree (except focal branches). Bars represent the probability of a fruit to be damaged according to the treatment (logistic regression model). For each bar, the confidence interval is provided.

The logistic regression analysis on data from the whole tree (focal branches excluded) showed similar results to those on the focal branches, highlighting a significant association between treatments and fruit status (damaged vs not damaged). Fruits from the ANs+/Ants+ trees were 3.47 times less likely to be damaged than the control group (ANs−/Ants+). Given the higher number of fruits with respect to the previous analysis, the differences between the control group and the other treatments (ANs+/Ants−; ANs−/Ants−) were also significant (see Table 4 and Figure 9). Specifically, the fruits from ANs−/Ants− trees, where ants were excluded, were 1.32 times more likely to be damaged than the control group (ANs−/Ants+). The addition of the random factor Tree-id to the model did not change the previous results.

## 4. Discussion

Results demonstrated that ANs can be used to manipulate ant presence on pear trees. Moreover, new data on the role of ants in pear agroecosystems and their impact as plant defenders were obtained. To the best of our knowledge, the list of ant species observed and their relative abundances may represent the first assessment of this kind in a pear orchard worldwide. The scarcity of arboreal-nesting species in the orchard is noteworthy, including the almost complete absence of ecologically and behaviorally dominant ants (see [104,111]) such as *Crematogaster scutellaris* (Olivier, 1792) (present in about 2.5% of the orchard trees) or *Lasius fuliginosus* (Latreille, 1798) (absent in the orchard), which were clearly more abundant in the surrounding areas. A lack of suitable nesting sites within the orchard trees may have contributed to this difference. The most representative species we found in the orchard are commonly recorded in the Padan Plain [112].

A fundamental result of this study is the success of ANs at attracting ants, helping to create a gradient in activity of ants among treatments. The existence of different ant activity levels among treatments through the use of ANs and sticky barriers was the fundamental premise to the success of the rest of the experiment, allowing an evaluation of how varying rates result in varying effects on other arthropods and on plants. Notably, these differences were successfully maintained throughout the season. An interesting high peak of ant activity, correlated with the presence of ANs, was recorded during the first week of monitoring (note that ANs were installed a week earlier). This could be explained as the result of a “novelty effect”, or of a latency before some degree of saturation of the colony “social stomach”, or by a change in dietary needs throughout the season (e.g., switching from carbohydrates to a protein-rich diet as more brood are produced) [113,114]. In any case, ANs should be further studied as possible tools to direct ant activity toward target areas over significantly long periods of time.

While a rich arthropod community was observed, most of the typical pests of pear orchards [89] were either absent or only few individuals were present. The San Jose scale, *Q. perniciosus*, against which the predatory activity of certain ants may be very effective [90], was absent. Similarly, *Cacopsylla* sp., which ants may effectively control or, according to others, favor [91,92,93], were only present in low numbers. Wasps (*Vespula* sp.) were the sole group showing significant differences in their presence on trees according to treatments: they were very poorly represented overall, but slightly more common in trees with ANs. This pattern seems to be the result of wasp interest in the artificial nectar (seldom observed in European hornets, *Vespa crabro* Linnaeus, 1761, too), which apparently grew throughout the period of the experiment. In a few instances, *Lasius* spp. were observed aggressively preventing the wasps from landing to drink at the AN dispensers they had occupied, and wasps usually aimed to land at dispensers without ants. Differences in the presence of other arthropod taxa were expected (e.g., [101,102,115]), but perhaps they would have required a much heavier sampling effort to be numerically appreciated. However, during the second month of the experiment, some cases of pest outbreaks were noted on a few trees with sticky barriers preventing ant presence. The fact that no increase in sap feeders was observed in conjunction with increased ant activity is notable. It is possible that ANs also decreased the importance of honeydew producers as nectary sources for ants, thus disrupting their protective tending behavior ([20,21,97,98,99,100]—but see also [116]). However, the ant–sap feeder association did not appear relevant in the study area.

Interesting differences were found regarding leaves among different treatments. The first two damage categories showed very similar trends, suggesting they both may be mainly attributed to the same pathogen (*V. pyrina*). In both cases, trees with a high ant activity were significantly less affected, which may indicate the role of ants in limiting the spread of this epiphytic fungus. Indeed, effects on plants by ant antimicrobial secretions have already been documented in several cases, but the ecological weight of these interactions—not to mention their possible applications in agriculture—is far from being understood and in need of investigation [17,48,79]. The ability of ants to reduce the damage by apple scab (*Venturia inequalis* (Cooke) G. Winter (1875)) was recently documented [48]. The other two damage categories had no significance in relation to ant activity. However, leaves were already quite damaged at the beginning of data sampling. As a consequence, an experiment specifically aimed at further evaluating the effects of an increase of ant presence on pear leaves may benefit from starting earlier in the season, in conjunction with the appearance of the first leaf gems.

Moreover, interesting data were obtained in relation to fruit production. Contrary to leaves, fruit were still in an early stage of development and mostly intact at the beginning of the experiment. By the time they were collected, a large percentage had been attacked by the codling moth (*C. pomonella*), which is considered to be one of the most important pear pests worldwide [89]. Such attacks tend to compromise both pulp and seeds, making fruit unsuitable for the food market and seed production, and damaging plant fitness. However, in trees in which ant activity was increased by ANs, the attacks were much less problematic. The two analyses carried out separately on focal branches and on the rest of the fruit production depicted very similar results. This suggests that, although ANs were placed on only two branches per tree, they increased ant activity on an unexpectedly larger area of the plant. We can speculate that ant activity may have limited the action of the codling moth through different, non-exclusive mechanisms, such as via a dissuasive effect on egg-laying activity (possibly mediated by semiochemicals), or by direct predation on the moth eggs and/or caterpillars. Ants are known to prey on both the eggs and larvae of different lepidopterans [48,71,117,118,119], and they also prey on other frugivorous insect larvae such as those of fruit flies [120,121,122]. Moreover, they may inhibit fruit fly egg deposition due to their secretions, and Tephritidae may even avoid landing on fruit previously exposed to ants [123]. Predation on the pupal stage in the soil may also have occurred (e.g., as concerns flies [124]). It is remarkable to note that none of the few biological control techniques currently employed against the codling moth are able to target eggs and larval stages [90,125,126,127]. Moreover, pesticide treatments against this pest face increasing difficulties due to the emergence of resistance mechanisms [128,129]. Additional investigation should clarify in detail how ants can affect the moths and their activity on plants, possibly leading to new control methods.

Both the ANs and nectar solution used during the experiment were very basic, and may be improved in the future, perhaps by further drawing inspiration from natural systems such as more elaborate nectar solutions with specific carbohydrates/nitrogen ratios or proteins, which could possibly enhance ant predatory attitude (e.g., [20,26,130,131]). Moreover, in comparison with a recent experiment of inoculation biological control employing ants in an apple orchard [48,71], we obtained somewhat similar beneficial results by using the native ant-fauna, while we did not witness sap feeders outbreaks.

## 5. Conclusions

Although a relatively simple protocol was employed, very encouraging results on fruit production and plant health were achieved, many of which solicit the need for additional investigation. Future efforts may focus on the mechanisms eventually adopted by ants to contain *V. pyrina* and *C. pomonella*, and on how the activity of different ant species may impact plants and their pests. Overall, significant results were observed when ant activity was increased due to the ANs, while no significant differences were detected between control trees in which ant activity was untouched and those where it was artificially reduced. Moreover, no negative side effects of AN use were found. In conclusion, these results further encourage studying the role of ants and the employment of ANs in agroecosystems and suggest it is worth continuing to explore unchecked possibilities in the development of new tools for CBC. 

## Figures and Tables

**Table 1:** List of the ant species most frequently observed on the trees during the experiment. For each species, the percentages of visited trees and of presence records are provided for Ants+ treatments.

Taxon	Subfamily, Tribe	Trees (*n =* 10)	Records (*n =* 450)	Counted Individuals (*n =* 450)
*Camponotus piceus* (Leach, 1825)	Formicinae, Camponotini	90%	11.3%	79
*Camponotus vagus* (Scopoli, 1763)	Formicinae, Camponotini	30%	0.9%	4
*Dolichoderus quadripunctatus* (Linnaeus, 1771)	Dolichoderinae, Dolichoderini	10%	6.4%	113
*Formica cunicularia* Latreille, 1798	Formicinae, Formicini	100%	12.9%	105
*Lasius niger* (Linnaeus, 1758)	Formicinae, Lasiini	40%	16.4%	335
*Lasius paralienus* Seifert, 1992	Formicinae, Lasiini	60%	22.4%	553
*Myrmica sabuleti* Meinert, 1861	Myrmicinae, Myrmicini	50%	2.0%	21
*Plagiolepis pygmaea* (Latreille, 1798)	Formicinae, Plagiolepidini	70%	9.1%	368
*Tapinoma subboreale* Seifert, 2012	Dolichoderinae, Tapinomini	10%	0.2%	1
*Temnothorax italicus* (Consani, 1952)	Myrmicinae, Crematogastrini	10%	0.2%	1

**Table 2:** List of the arthropods scanned weekly on the two focal branches from the beginning of July until the end of August 2018 (nine weeks). “Total” represents the total number of individuals recorded during the whole sampling period. In *p*, statistically significant results are highlighted in bold. In “Treatment”, treatments with the same letter are not statistically different. In “Mean” and “SE”, results refer to *n =* 90.

Class	Order	Family/Species	Total	H_(3)_	*p*	Treatment	Mean	SE
Arachnida	Araneae		29	6.268	0.099	ANs+/Ants+	0.04	0.02
						ANs−/Ants+	0.03	0.02
						ANs+/Ants−	0.14	0.05
						ANs−/Ants−	0.10	0.03
Insecta	Coleoptera		33	1.759	0.624	ANs+/Ants+	0.07	0.03
						ANs−/Ants+	0.09	0.07
						ANs+/Ants−	0.13	0.05
						ANs−/Ants−	0.08	0.03
	Dermaptera		1	-	-	-	-	-
	Diptera		35	0.727	0.867	ANs+/Ants+	0.07	0.03
						ANs−/Ants+	0.13	0.05
						ANs+/Ants−	0.09	0.03
						ANs−/Ants−	0.10	0.04
	Hemiptera	*M. pruinosa*	65	3.402	0.334	ANs+/Ants+	0.03	0.02
						ANs−/Ants+	0.19	0.08
						ANs+/Ants−	0.17	0.06
						ANs−/Ants−	0.33	0.22
		*S. pyri*	192	9.094	0.028	ANs+/Ants+	0.00	0.00
						ANs−/Ants+	0.40	0.20
						ANs+/Ants−	1.73	1.19
						ANs−/Ants−	0.00	0.00
		others	32	2.348	0.503	ANs+/Ants+	0.08	0.03
						ANs−/Ants+	0.08	0.03
						ANs+/Ants−	0.03	0.01
						ANs−/Ants−	0.08	0.03
	Hymenoptera	*Vespula* sp.	37	37.749	<0.001	(A)ANs+/Ants+	0.14	0.04
						(B)ANs−/Ants+	0.00	0.00
						(A)ANs+/Ants−	0.27	0.06
						(B)ANs−/Ants−	0.00	0.00
		others	4	-	-	-	-	-
	Lepidoptera	*H. cunea*	631	9.050	0.029	ANs+/Ants+	0.00	0.00
						ANs−/Ants+	0.00	0.00
						ANs+/Ants−	0.00	0.00
						ANs−/Ants−	7.01	4.92
		others	4	-	-	-	-	-
	Neuroptera	Chrysopidae	7	-	-	-	-	-
		Chrysopidae (eggs)	40	6.642	0.503	ANs+/Ants+	0.02	0.02
						ANs−/Ants+	0.10	0.04
						ANs+/Ants−	0.11	0.04

**Table 3:** Logistic regression of treatments (ANs+/Ants+; ANs−/Ants+; ANs+/Ants−; ANs−/Ants−) on fruit status (damaged vs not damaged). The coefficients of treatments are contrasts with the control group (ANs−/Ants+). ** *p* < 0.01; *** *p* < 0.0001.

Variable	B	SE	Odds Ratio	Sig.
Constant	−1.04	0.25	−4.18	<0.001 ***
ANs+/Ants+	−3.06	1.04	−2.95	0.003 **
ANs+/Ants−	−0.80	0.41	−1.96	0.05
ANs−/Ants−	−0.12	0.43	−0.29	0.76
χ^2^	22.18, *df* = 3, *p* < 0.001 ***			

**Table 4:** Logistic regression of treatments (ANs+/Ants+; ANs−/Ants+; ANs+/Ants−; ANs−/Ants−) on fruit status (damaged vs not damaged). The coefficients of treatments are contrasts with the control group (ANs−/Ants+). * *p* < 0.05; ** *p* < 0.01; *** *p* < 0.0001.

Variable	B	SE	Odds Ratio	Sig.
Constant	−1.93	0.08	0.15	<0.001 ***
ANs+/Ants+	−1.34	0.13	0.26	<0.001 ***
ANs+/Ants−	−0.23	0.11	0.80	0.03 *
ANs−/Ants−	0.33	0.11	1.39	0.002 **
χ^2^	231.09, *df* = 3, *p* < 0.001 ***			

## Supplementary Materials

The following are available online at https://www.mdpi.com/2075-4450/11/2/129/s1, Table S1: List of ant species recorded during the preliminary assessment of the ant fauna of the study area, Table S2: List of arthropods (ants excluded) observed on the trees during the experiment, Table S3: Fruit production of the experimental trees and damage inflicted by the codling moth caterpillar (*Cydia pomonella*).
