# Supplementary material for: New Tools for Conservation Biological Control: Testing Ant-Attracting Artificial Nectaries to Employ Ants as Plant Defenders
Source: Insects. 2020 Feb 17;11(2):129. doi: 10.3390/insects11020129 (PMC7074267; doi:10.3390/insects11020129)
Supplement: Supplementary file 1 [file insects-11-00129-s001.pdf]

Table S1. List of ant species recorded during the preliminary assessment of the ant fauna of the study area. Species marked with an asterisk were not recorded within the orchard, but exclusively at its external margins.

Aphaenogaster subterranea (Latreille, 1798) *
Camponotus lateralis (Olivier, 1792)
Camponotus piceus (Leach, 1825)
Camponotus vagus (Scopoli, 1763)
Colobopsis truncata (Spinola, 1808)
Crematogaster scutellaris (Olivier, 1792)
Dolichoderus quadripunctatus (Linnaeus, 1771)
Formica cunicularia Latreille, 1798
Lasius emarginatus (Olivier, 1792) *
Lasius fuliginosus (Latreille, 1798) *
Lasius niger (Linnaeus, 1758)
Lasius paralienus Seifert, 1992
Messor ibericus Santschi, 1931
Myrmica sabuleti Meinert, 1861
Polyergus rufescens (Latreille, 1798)
Solenopsis fugax (Latreille, 1798)
Tapinoma subboreale Seifert, 1992
Temnothorax italicus (Consani, 1952)
Tetramorium sp. (*caespitum*-complex)

Table S2. List of arthropods (ants excluded) observed on the trees during the experiment. Species not observed during weekly investigation of the focal branches but instead during other activities are marked with an asterisk.

Taxon	Order, Family
(unidentified) *	Acarina
Araneus diadematus Clerck, 1757 *	Araneae, Araneidae
Cyclosa sp. *	Araneae, Araneidae
Others (unidentified)	Araneae, Araneidae
Clubiona sp. *	Araneae, Clubionidae
Cheiracanthium sp.	Araneae, Eutichuridae
(unidentified) *	Araneae, Gnaphosidae
Oxyopes sp.	Araneae, Oxyopidae
Palpimanus gibbulus Dufour, 1820 *	Araneae, Palpimanidae
Heliophanus sp. *	Araneae, Salticidae
Others (unidentified)	Araneae, Salticidae
Micrommata ligurina (C.L. Koch, 1845) *	Araneae, Sparassidae
Thomisus onustus Walckenaer, 1805 *	Araneae, Thomisidae
Xysticus sp. *	Araneae, Thomisidae
Others (unidentified)	Araneae, Thomisidae
Zoropsis sp. *	Araneae, Zoropsidae
Others (unidentified)	Araneae
Argiolus sp.	Coleoptera, Buprestidae
Alticinae *	Coleoptera, Chrysomelidae
Chilocorus bipustulatus (Linnaeus, 1758)	Coleoptera, Coccinellidae
Harmonia axyridis (Pallas, 1773)	Coleoptera, Coccinellidae
Hippodamia variegata (Goeze, 1777)	Coleoptera, Coccinellidae
Propylea quatuordecimpunctata (Linnaeus, 1758)	Coleoptera, Coccinellidae
(unidentified) *	Coleoptera, Curculionidae
(unidentified) *	Coleoptera, Elateridae
Lucanus cervus (Linnaeus, 1758) *	Coleoptera, Lucanidae
Protaetia sp.	Coleoptera, Scarabeidae
Others (unidentified Cetoniinae)	Coleoptera, Scarabeidae
Others (unidentified)	Coleoptera
(unidentified) *	Collembola
Forficula sp.	Dermaptera, Forficulidae
(unidentified)	Diptera, Asilidae

Drosophila sp.	Diptera, Drosophilidae
(unidentified)	Diptera, Muscidae
Sarcophaga sp.	Diptera, Sarcophagidae
Myathropa floraea (Linnaeus, 1758)	Diptera, Syrphidae
(unidentified)	Diptera, Tachinidae
Others (unidentified Brachycera)	Diptera
Others (unidentified Nematocera)	Diptera
(unidentified) *	Hemiptera, Aphidiidae
(unidentified) *	Hemiptera, Coreidae
Pilophorus sp.	Hemiptera, Myridae
Others (unidentified)	Hemiptera, Myridae
Halyomorpha halys Stål, 1855 *	Hemiptera, Pentatomidae
Nezara viridula (Linnaeus, 1758)	Hemiptera, Pentatomidae
Raphigaster nebulosa (Poda, 1761) *	Hemiptera, Pentatomidae
(unidentified) *	Hemiptera, Reduviidae
Stephanitis pyri (Fabricius, 1775)	Hemiptera, Tingidae
Others (unidentified Heteroptera)	Hemiptera
Cercopidea	Hemiptera,
Metcalfa pruinosa (Say, 1830)	Hemiptera, , Flatidae
Cacopsylla sp.	Hemiptera, , Psyllidae
parasitoids (unidentified)	Hymenoptera
Vespa crabro Linnaeus, 1758	Hymenoptera, Vespidae
Vespula sp.	Hymenoptera, Vespidae
Caliroa cerasi (Linnaeus, 1758)	Hymenoptera, Tenthredinidae
Cossus cossus (Linnaeus, 1758)	Lepidoptera, Cossidae
Hyphantria cunea (Drury, 1773)	Lepidoptera, Erebidae
Brintesia circe (Fabricius, 1775) *	Lepidoptera, Nymphalidae
Melitaea sp.	Lepidoptera, Nymphalidae
Iphiclides podalirius (Linnaeus, 1758) *	Lepidoptera, Papilionidae
Cydia cf. *pomonella* (Linnaeus, 1758) *	Lepidoptera, Tortricidae
(unidentified) *	Lepidoptera, Geometridae
Others (unidentified)	Lepidoptera
(unidentified)	Neuroptera, Chrysopidae
(unidentified) *	Neuroptera, Mantispidae
Phaneroptera nana Fieber, 1853 *	Orthoptera, Tettigoniidae
Others (unidentified) *	Orthoptera

Tab. S3. Fruit production of the experimental trees and damage inflicted by the codling moth caterpillar (*Cydia pomonella*). Results are shown according to Damaged Fruits percentages (increasing gradient). In *Treatment* column, the ANs+/Ants+ treatment (high ant activity) is highlighted in bold.

Tree	Pear Variety	Treatment	Date of harvesting	Harvested fruits (N°)	Damaged Fruits (%)
1	Giallo	**ANs+/Ants+**	17/07/2018	271	0,37
2	Ducal	**ANs+/Ants+**	02/10/2018	95	3,16
5	Spadon	**ANs+/Ants+**	02/10/2018	177	3,39
3	Russet	**ANs+/Ants+**	02/10/2018	1735	3,92
4	Estif	**ANs+/Ants+**	03/08/2018	465	4,52
15	Nobile	ANs+/Ants-	02/10/2018	676	5,62
7	Nobile	ANs-/Ants+	02/10/2018	463	6,05
12	Giallo	ANs+/Ants-	17/07/2018	557	8,08
9	Ingurien	ANs-/Ants+	06/08/2018	529	9,07
11	Ragul	ANs+/Ants-	02/10/2018	506	9,68
14	Ducal	ANs+/Ants-	02/10/2018	65	10,77
16	Ingurien	ANs-/Ants-	06/08/2018	482	11,41
17	Nobile	ANs-/Ants-	16/09/2018	479	11,48
18	Cavicchione	ANs-/Ants-	02/10/2018	421	12,83
10	Delacuatorta	ANs-/Ants+	02/10/2018	264	15,53
8	Ducal	ANs-/Ants+	02/10/2018	114	18,42
13	Ingurien	ANs+/Ants-	06/08/2018	390	23,33
20	Ducal	ANs-/Ants-	02/10/2018	98	38,78
6	Decorè	ANs-/Ants+	02/10/2018	86	67,44
19	Decorè	ANs-/Ants-	02/10/2018	97	68,04
